# Risk factors for infection by oncogenic human papillomaviruses in HIV-positive MSM patients in the ART era (2010–2016)

**DOI:** 10.1097/MD.0000000000008109

**Published:** 2017-09-29

**Authors:** Carmen Hidalgo-Tenorio, Concepción Gil-Anguita, Jessica Ramírez-Taboada, Javier Esquivias, Miguel A. López-Ruz, Omar Mohamed Balgahata, Rosario Javier-Martinez, Juan Pasquau

**Affiliations:** a Department of Infectious Diseases; b Department of Pathology, Virgen de las Nieves University Hospital, Granada; c Unit of Infectious Diseases, “University Hospital Complex”, Jaen, Spain.

**Keywords:** high-risk human papillomavirus, HIV, HSIL, men who have sex with men, squamous cell carcinoma of anus

## Abstract

Squamous cell carcinoma of anus (SCCA) is one of the most frequent non-AIDS-defining diseases in HIV patients, mainly in men who have sex with men (MSM), and it is associated with human papillomavirus (HPV) infection.

To determine the prevalence of high-risk HPV (HR-HPV) genotypes, premalignant lesions (HSIL) and SCCA in a cohort of HIV-positive MSM; to study the distribution of HPV genotypes according to anal histology results; and to analyze risk factors for this infection.

This prospective single-center study was conducted between May 2010 and September 2016. At the study visit, cotton swabs were used to collect anal samples for cytology study in ThinPrep Pap Test liquid medium (Thin Prep Processor 2000, Hologic Corp, USA), and for HPV PCR (Linear Array HPV Genotyping Test). After, high-resolution anoscopy (HRA) (Zeiss 150 fc^©^) was carried out. Logistic regression analysis was performed to identify risk factors for HR-HPV infection.

The study included 319 patients, with mean age of 36.7 years; HR-HPV was detected in 81.3%. The prevalence of HSIL was 13.5% and SCCA was 0.3%. With regard to the distribution of HPV genotypes according to histology results, HPV 16 was the most frequent genotype in normal anal mucosa (26.7%), in LSILs (36.9%), and in HSILs (38%). In multivariate analysis, CD4 nadir < 200 cells/μL was the factor associated with infection by HR-HPV (OR 3.66, 95% CI 1.05%–12.75%).

HIV-positive MSM showed a high prevalence of HSIL+ lesions and of infection by oncogenic HPV, which appears to be favored by a deficient immune system. HPV 16 was the most frequently isolated genotype in anal mucosa, regardless of lesion type.

## 1 Introduction

Human papillomavirus (HPV) infection is the most prevalent sexually transmitted disease worldwide and is more frequent among females, men who have sex with men (MSM), and immunodepressed patients (HIV, kidney transplant).^[1]^ Infection with HPV, especially with oncogenic or high-risk HPV (HR-HPV), has been associated with squamous cell carcinoma of anus (SCCA) and cervical cancer.^[2]^ SCCA is rare in the general population, with an incidence of around 1.8 cases per 100,000 persons/year.^[3]^ However, it is one of the most frequent non-AIDS-defining neoplasms in HIV patients,^[4]^ especially in MSM, with a very high incidence of up to 144 cases per 100,000 persons/year,^[5]^ although survival rates are similar to those in the general population.^[6]^ For this reason, it is of major interest to establish the prevalence of HPV infection in these patients as well as to study the distribution of HPV genotypes in relation to histological lesion type and identify predictive factors for this infection.

Data on the distribution of HIV genotypes related to anal mucosa histology have only been published on patients with abnormal cytology and with high-resolution anoscopy (HRA) findings suggestive of dysplastic lesion.^[7]^ Moreover, the sensitivity of anal cytology to screen for high-grade squamous intraepithelial lesion (HSIL)+ lesions (HSIL, CA) is variable and limited, resulting in an appreciable percentage of under-diagnosed patients, as demonstrated by some studies.^[8–10]^

The objectives of this study were: to establish the prevalence and distribution of HPV genotypes in a population of HIV-positive MSM according to anal mucosa biopsy results; to determine the prevalence of HSIL+ anal lesions; and to identify the risk factors for HPV infection.

## 2 Patients and methods

### 2.1 Design

An observational, prospective, single-center study was conducted in a cohort of consecutive HIV-positive MSM enrolled between May 2010 and September 2016 in a screening, diagnosis, treatment, and follow-up program for dysplastic anal mucosa lesions in the Infectious Diseases Department of a 3rd-level hospital in Southern Spain. This study was approved by the ethics committee of the hospital. Epidemiological, clinical, and analytical data were gathered and treated following the data protection law in force (Organic Law 15/1999, 13 December, of Personal Data Protection).

Inclusion criteria were: age ≥18 years, self-reported MSM, HIV positivity, and signing of informed consent to study participation. Exclusion criteria were: female sex, self-reported heterosexuality, and history of anal canal neoplasm.

At the visit, the variables collected were: age, history of perianal-genital condyloma, number of sexual partners as anal receptor during previous 12 months, utilization of condoms, smoking habit, alcohol consumption in standard drinking units (SDUs), parenteral drug addiction, nationality, educational level, months since HIV diagnosis, and HIV stage according to the CDC classification; months receiving antiretroviral therapy (ART), virological failure (viral-RNA >50 copies/mL in at least 2 tests during the previous 6 months); and receipt of any concomitant treatment, and the presence of other infections, including chronic liver disease from hepatitis B (HBV) or hepatitis C (HCV) virus, syphilis, other sexually transmitted diseases, and latent, treated, or active tuberculosis infection. Analytical data were also gathered on CD4 nadir cell count, CD4 lymphocyte count and viral load at the HIV diagnosis, and on CD4, CD8, and viral load at the study visit.

During the study visit, 2 anal canal samples were taken using 2 cotton swaps impregnated in physiological saline: one for HPV detection and genotyping by qualitative polymerase chain reaction (PCR) (Linear Array HPV Genotyping Test) using a “GeneAmp PCR System 9700” thermocycler (Applied Biosystems, Roche, Switzerland); and the other for the cytology study; both samples were immersed in thin layer liquid medium for application of the thin-layer cytology technique using a ThinPrep Pap Test liquid medium (Thin Prep Processor 2000, Hologic Corp, Marlborough, MA). Both samples were sent to the pathology laboratory, and the results of both techniques were evaluated by a single pathologist (JE). Genotypes 16, 18, 26, 31, 33, 35, 39, 45, 51 to 53, 56, 58, 59, 66, 68, 73, and 82 were considered HR-HPVs, classifying genotypes 39, 45, 59, and 68 as subspecies of HPV 18, and genotypes 31, 33, 35, 52, 58, and 67 as subspecies of HPV 16. Genotypes 6, 11, 34, 40, 42 to 44, 54, 55, 57, 61, 70 to 72, 81, 83, 84, and 89 were considered low-risk HPVs (LR-HPVs).^[11]^ Between 4 and 12 weeks after the study visit, patients underwent HRA with a Carl Zeiss 150 fc^©^ colposcope (Carl Zeiss, Oberkochen, Germany). After digital rectal exam, a disposable transparent anoscope was introduced for the installation of 5 mL acetic acid and left in place for around 3 minutes, examining the mucosa after its removal; next, 5% Lugol iodine was instilled and left for 1 minute, followed by a 2nd anoscopy. Samples were taken from apparently normal mucosa in the 4 quadrants and from areas with Lugol-negative acetowhite lesions. Biopsies were conducted using an endoscopic retrograde cholangiopancreatography (ERCP) catheter.

The cytology study used Bethesda classification,^[12]^ which divides lesions into atypical squamous cells (ASC), ASC-high, low-grade squamous intraepithelial lesion (LSIL), and HSIL. The histology study used the classification of the Lower Anogenital Squamous Terminology (LAST) Standardization Project for HPV, which divides lesions into LSIL (AIN1/condyloma), HSIL (AIN2, AIN3), and SCCA.^[13]^ These studies were always conducted by the same pathologist (JE).

Definition of variables:

Abnormal cytology was considered to include ASC of unknown significance (ASCUS), LSIL, or HSIL.

In the histology study, “HSIL+ anal lesions” were considered to range from HSIL to SCCA, and “LSIL+ anal lesions” were from LSIL to SCCA.

Patients were considered late-presenters when the CD4 cell count was <200 cells/μL at HIV diagnosis.

### 2.2 Statistical analysis

Means, standard deviations, medians, and percentiles were calculated for quantitative variables and absolute frequencies with 95% confidence interval for qualitative variables. Prevalence of HPV, anal mucosa cytology, and histology findings were calculated with 95% confidence interval.

Bivariate tests were conducted to study the relationship between possible risk factors and HR-HPV infection. After evaluation of the distribution of variables using the Kolmogorov–Smirnov test, the Student *t* test for independent samples was applied for quantitative variables that were normally distributed and the Mann–Whitney *U* test for those that were not, while the Pearson chi-square test was used for qualitative variables that were normally distributed and Fisher test for those that were not. The Kappa index was used to analyze the concordance between HR-HPV PCR and biopsy results, considering k < 0.20 poor, 0.21 to 0.40 weak, 0.41 to 0.60 moderate, 0.61 to 0.80 good, and 0.81 to 1.00 very good agreement, following Landis and Koch test.^[14]^

Multivariate logistic regression analysis was performed, based on Freeman formula [n = 10∗(k + 1)].^[15]^ The model included variables that were statistically significant in bivariate analyses and those considered clinically relevant, including: age, age >50 years, employment status, number of partners during the previous 12 months, age at first sexual relationship, use of condom, the presence of perianal or genital warts, smoking habit, history of syphilis or other sexually transmitted infection, CD4 nadir, CD4 nadir <200 cells/μL, CD4 count at HIV diagnosis, CD4 count and viral load at the time of the study, receipt of ART, time with ART, and time since HIV diagnosis. A stepwise method was used, with an entry *P*-value of .05 and exit *P*-value of .10 at each step. The Hosmer–Lemeshow test was applied to analyze the goodness of fit of the model. SPSS 20.0 (IBM plc, Chicago, IL) was used for the statistical analyses. *P* < .05 was considered significant in all tests.

## 3 Results

### 3.1 Baseline characteristics of participants

A total of 319 HIV-positive MSM were recruited between May 2010 and September 2016. The mean age was 36.7 years and the mean CD4 nadir was 361.1 cells/μL; CD4 nadir <200 cells/μL was recorded in 24% of participants. The median time with HIV was 31 months (interquartile range [IQR]: 9–91 months); 86.2% had been on ART for a median of 19 months (IQR: 6–65.5 months), with a mean of CD4 698.5 cells/μL, and only 1.8% were in virological failure. Table 1 exhibits results for the remaining study variables.

**Table 1:**
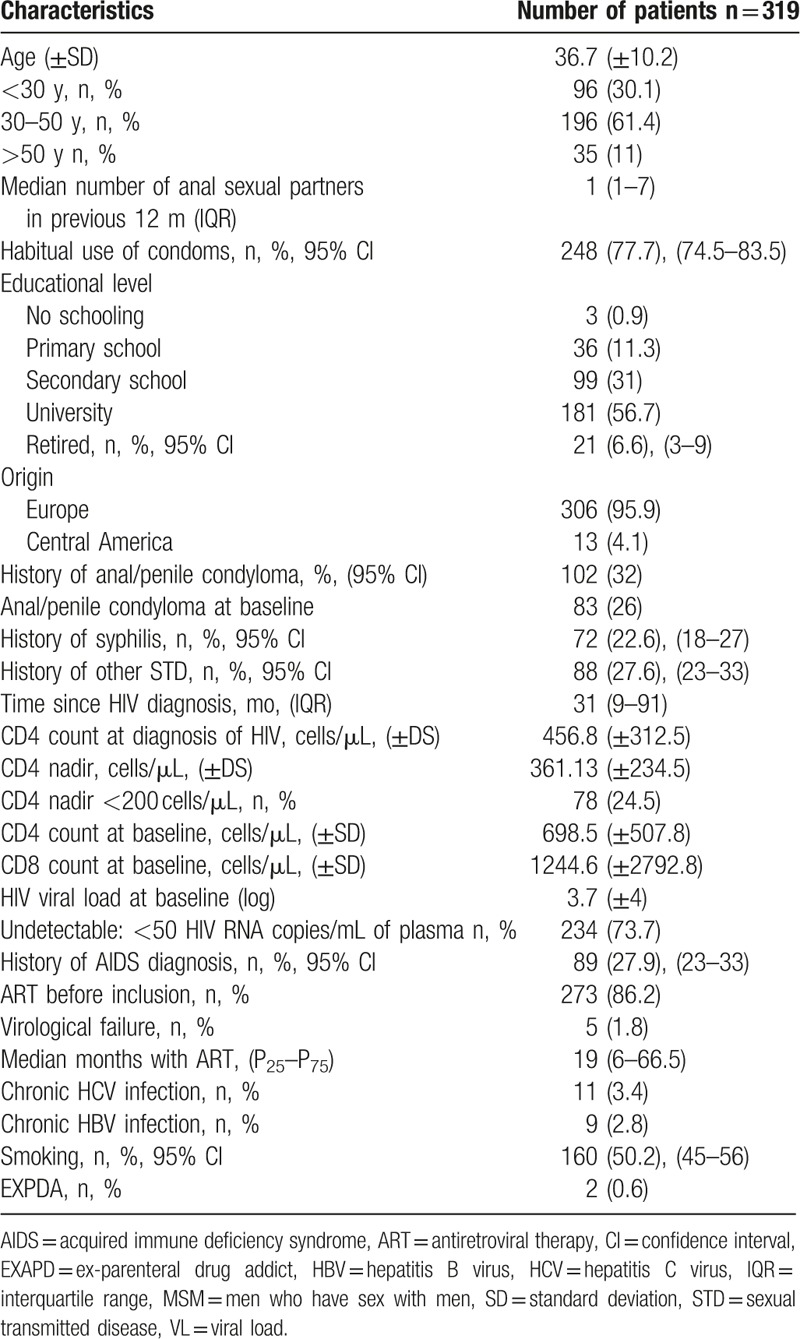
Baseline characteristics of the HIV-positive MSM patients.

### 3.2 Results of cytology, HPV PCR, and anal mucosa biopsy

**Figure 1:**
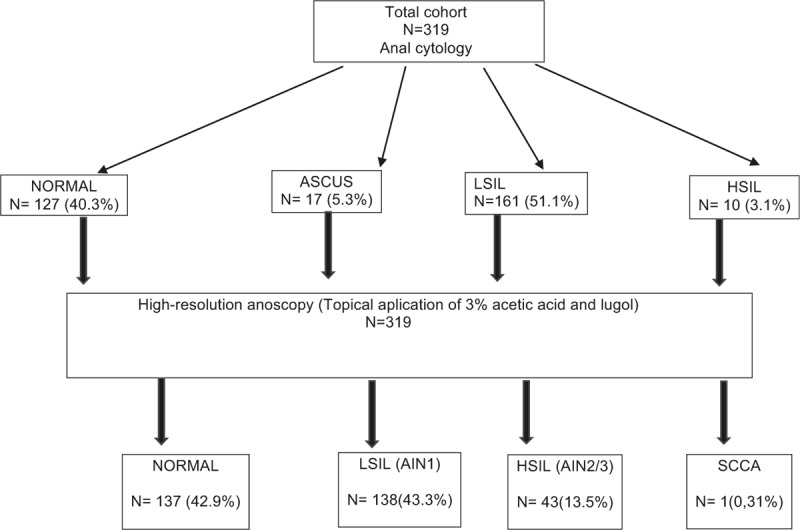
Flow chart of patients through the study.

Anal cytology results were normal in 40.3% of participants and showed LSIL in 51.1%, HSIL in 3.1%, and ASCUS in 5.3% (Fig. 1).

According to anal mucosa HPV PCR results, 311 of the 319 samples analyzed were valid. HR HPV genotypes were detected in 81.9% of these (95% CI 78–86), with a median of 2 (1–3); LR-HPV genotypes were detected in 71.1% (95% CI 66–76), with a median of 1 (0–2); and simultaneous infection with low- and high-risk genotypes was detected in 59.5% (95% CI 54–65). The most frequently isolated LR-HPVs in anal mucosa were HPVs 6 (17.7%), 11 (18%), 42 (18%), 61 (9.4%), and 81 (11.3%); and the most frequently isolated HR-HPVs were HPVs 16 (32.9%), 18 (14.1%), 31 (14.5%), 45 (13.5%), 51 (16.4%), 55 (15.7%), 66 (12.9%), and 68 (13.2%) (Table 2).

**Table 2:**
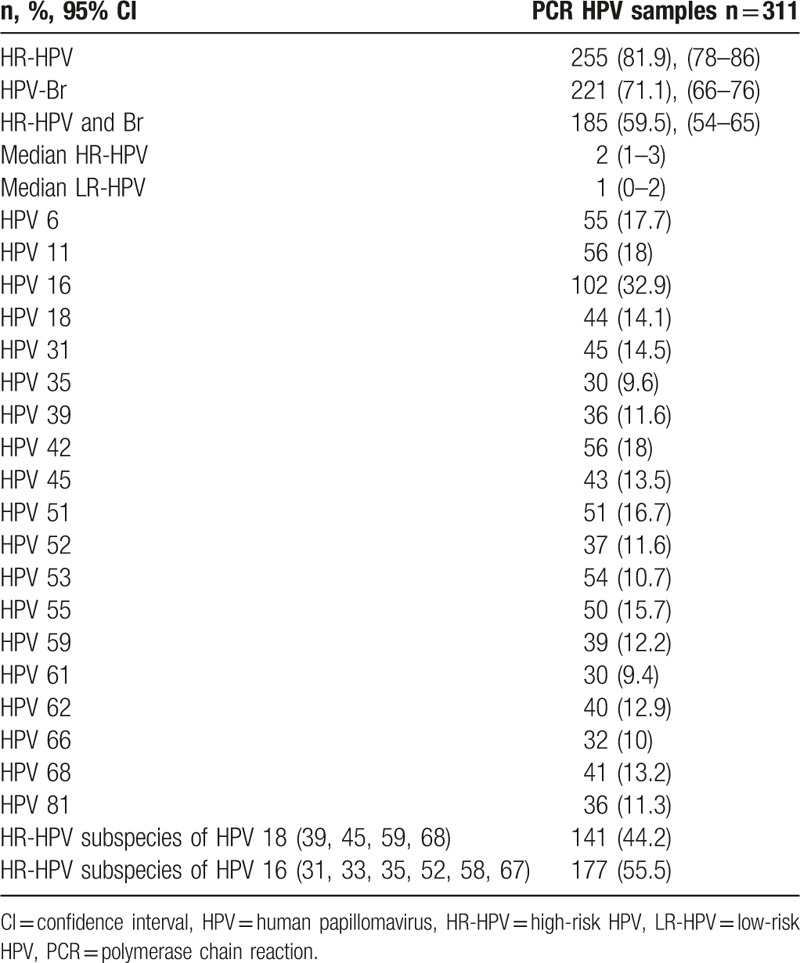
Prevalence of HPV in anal mucosa.

Anoscopy findings were normal in 42.9% of the 319 participants and showed LSIL in 43.3%, HSIL in 13.5%, and SCCA in 0.3% (Fig. 1).

Anal mucosa biopsy findings demonstrated that 11 (25%) of the patients with normal cytology were HSIL+ (10 HSIL and 1 SCCA), whereas no HSILs+ were found in the 25 (7.8%) patients with normal cytology and negative HR-HPV PCR (Table 3). Among the 43 patients with biopsy findings of HSIL, 76.7% had evidenced abnormal cytology; among these, lesions were low-grade in 65.1%, high-grade in 11.6%, and of uncertain significance in 2.3% (Table 3). Finally, 88.8% of patients with high-grade lesions in the histologic study showed infection by high-risk virus in anal canal mucosa (Table 3).

**Table 3:**
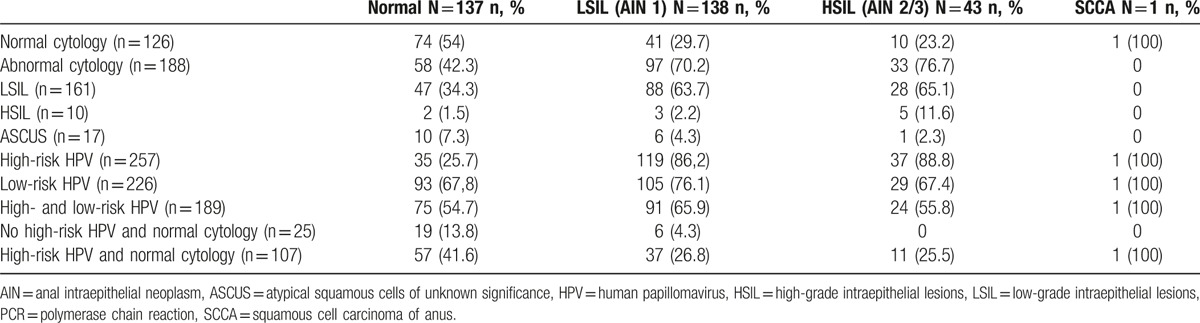
Number and proportion of histological diagnoses stratified by anal cytology and HPV PCR results.

### 3.3 HPV PCR distribution as a function of histology

**Figure 2:**
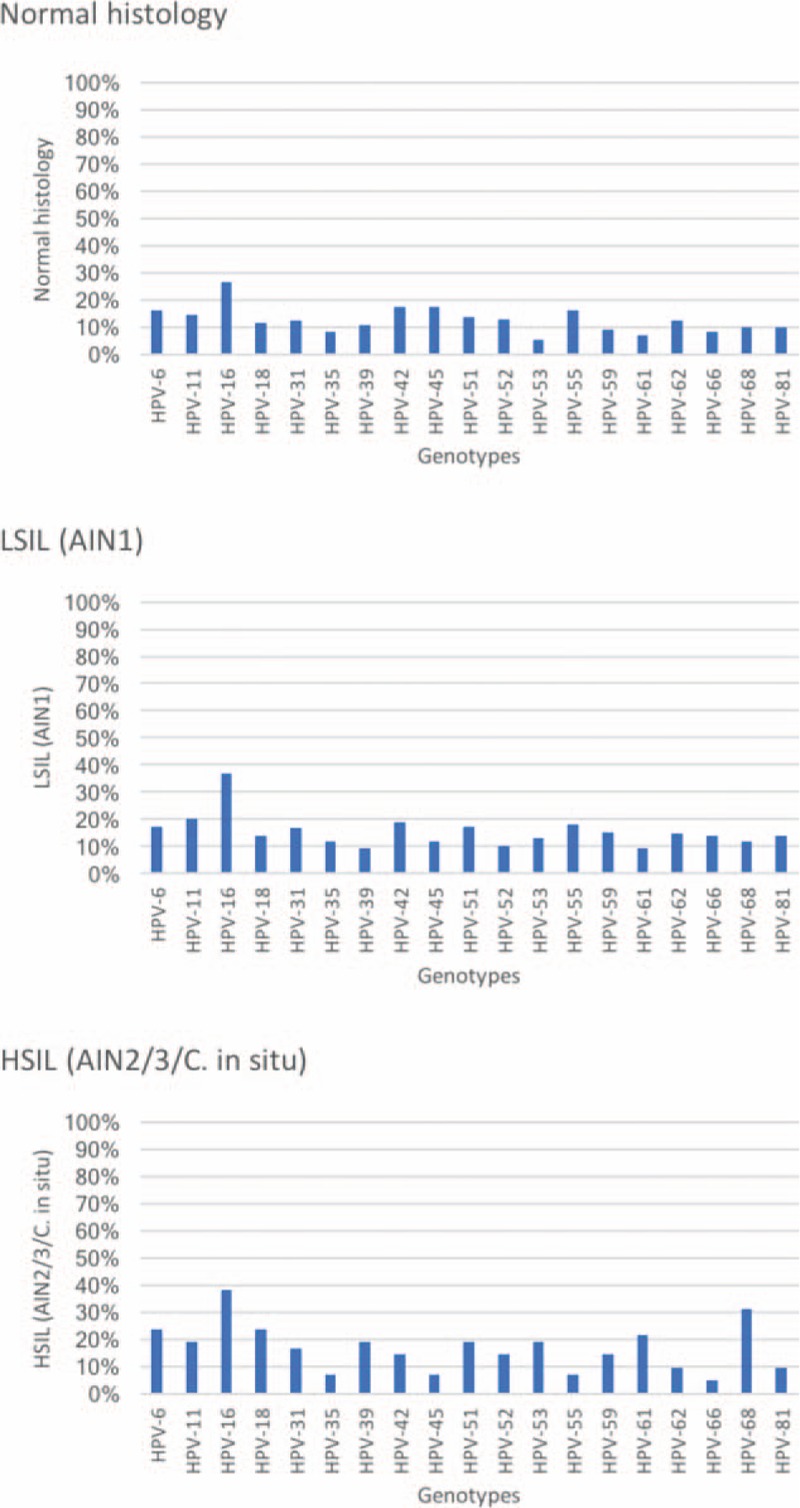
Distribution and prevalence of HPV genotypes in HIV-positive MSM patients by histological diagnosis. HPV = human papillomavirus, MSM = men who have sex with men.

The most frequently isolated HPV genotypes were HPVs 6 (16%), 16 (27%), 45 (18%), and 55 (16%) in normal biopsies; HPVs 16 (37%), 11 (20%), 42 (19%), and 55 (18%) in LSIL biopsies; and HPVs 6 (24%), 16 (38%), 18 (24%), and 68 (31%) in HSIL biopsies. Figure 2 depicts the distribution of the remaining genotypes.

Infection by genotype 16 was significantly associated with LSIL+ lesions (37.4% vs 26.7% of normal biopsies; *P* = .047; Kappa = 0.09; RR = 1.64; 95% CI 1.004–2.68). A significant association with HSIL+ lesions was found for genotype 68 (30.2% vs 10.5%, respectively, *P* = .0001; Kappa = 0.202, *P* = .0001; RR 3.71; 95% CI 1.7–7.9), and genotype 53 (20.9% vs 9.3%, *P* = .03; Kappa: 0.217, *P* = .024; RR = 2.57 (95% CI 1.1–5.97).

### 3.4 Predictive factors of infection by HR-HPV

In bivariate analyses, the following factors emerged as protective factors against infection by oncogenic virus: older age (36.1 vs 39.2 years, *P* = .0001; crude OR: 4.5), retirement (5% vs 14.3%; *P* = .017; crude OR: 0.32), higher CD4 cell count (675.5 vs 828.7 cells/μL; *P* = .04; crude OR: 1), and viral load <50 copies/mL (70.9 vs 85.4%, *P* = .047; crude OR: 0.47). Remaining findings are listed in Table 4.

**Table 4:**
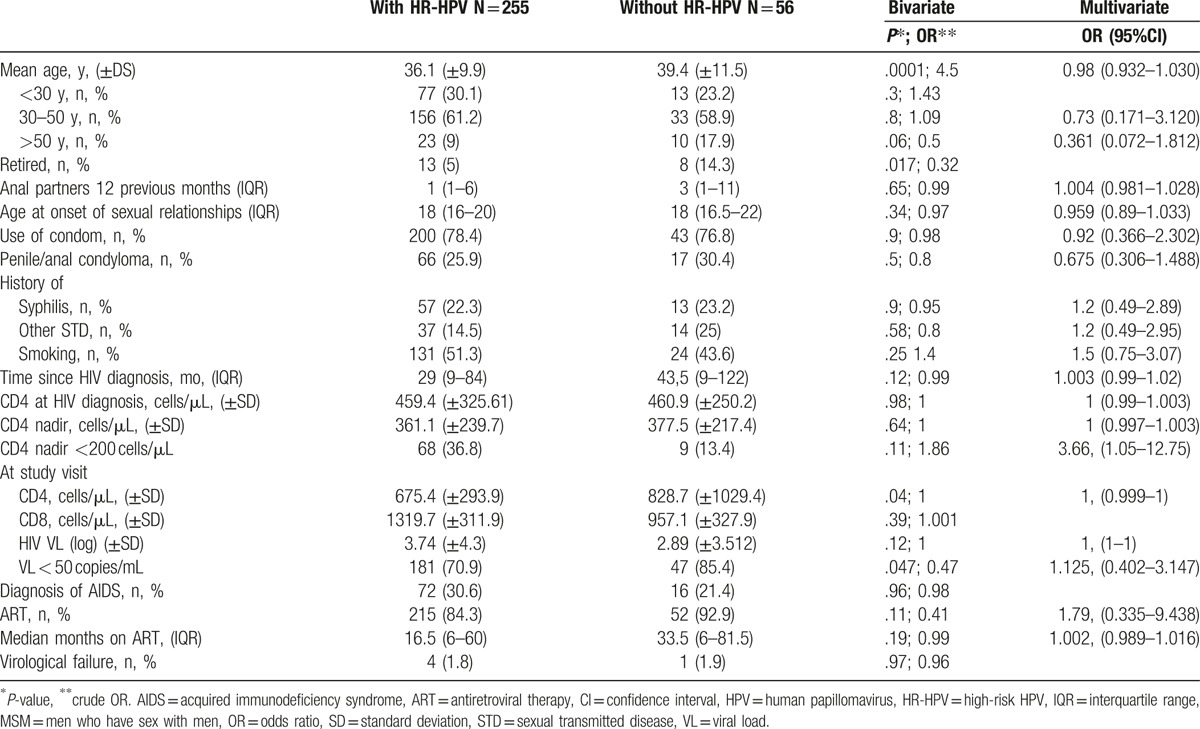
. Risk factors for infection by HR-HPV genotype in MSM patients with HIV bivariate and multivariate analyses.

In the multiple logistic regression analysis, the only significant risk factor for infection by oncogenic HPV was CD4 nadir <200 cells/μL (OR: 3.66; 95% CI 1.05–12.75) (Table 4).

## 4 Discussion

A high prevalence of HPV infection in anal mucosa was observed in this cohort of MSM patients with HIV. HR-HPV genotypes were detected in 81.9% of the patients and LR-HPV genotypes in 71.1%, while both HR-HPV and LR-HPV genotypes were identified in 59.5%. The most frequently isolated genotype was HPV 16, followed by the low-risk genotypes 42, 11, and 6. An elevated prevalence of anal infection by HR- and LR-HPV was previously reported in MSM patients with HIV^[16]^ and was significantly associated with cytological abnormalities.^[17]^ However, a lower prevalence of oncogenic viruses has been reported in Asian seropositive MSM.^[18,19]^ This difference may be attributable to the lower age of our patients (36.7 ± 10.2 years) in comparison to the former study (46 years, range 37–56),^[18]^ or to the different techniques used to detect HPV in anal mucosa.^[19]^

With respect to the distribution and prevalence of genotypes according to histological findings, the most frequent low-risk genotypes were: HPV 6 in patients with histologically normal mucosa, HPVs 11 and 42 in LSIL, and HPV 6 in HSIL. The most frequent high-risk genotypes were HPVs 16, 45, and 55 in normal mucosa, HPVs 16 and 55 in LSIL, and HPVs 16, 18, and 68 in HSIL. The most prevalent genotype in all mucosa types was HPV 16, which was significantly associated with LSIL+ lesions. HPVs 18 and 53, less prevalent than HPV 16, were associated with the presence of HSIL+ lesions. In previous studies of seropositive and seronegative patients, the most prevalent HR-HPV in anal mucosa was genotype 16, regardless of sex or sexual orientation,^[19,20]^ and this genotype has also been associated with high-grade lesions in seropositive MSM individuals and with other oncogenic viruses, for example, HPV 51.^[21]^

These results are similar to published data on the prevalence of HPV genotypes in HIV-positive MSM according to histological findings, although these showed some differences, even including the absence of LR-HPV 11 and HR-HPV 45, among others.^[7]^ In addition, the previous study was limited by an important selection bias, given that the distribution of HPV genotypes was only analyzed in individuals with abnormal cytology and pathological HRA findings, with biopsies only being ordered for 94 (19.4%) of the 483 participants.^[7]^

In the present study of HIV-positive MSM with, HSILs were detected in 13.5% and SCCA in 0.3%, that is, 1 out of 7 patients had these lesions, similar to a recent report on seropositive Spanish MSM in the ART era.^[21,22]^ Around one-quarter of HSIL+ lesions were in patients whose anal mucosa cytology had been normal. Hence, if the screening for dysplastic lesions had been based on cytology alone, as recommended by the scientific societies, 10 of the patients with HSIL and the patient with SCCA would have been undiagnosed and untreated. On the other hand, we highlight that no HSIL+ lesions were detected in patients with normal cytology and negative HR-HPV PCR. Anal cytology showed only moderate sensitivity in the present series. The cytology was previously observed to be more useful in cases with high-grade lesions, a larger number of involved quadrants, HIV positivity and CD4 cell count <200 cells/μL.^[23]^ In contrast, the majority of our patients had an excellent immune status, which predicts low sensitivity for cytology results. It therefore appears essential to optimize the screening of precursor and anal cancer lesions in HIV-infected MSM. We therefore propose that HPV PCR is performed in patients with normal cytology, because a negative result would rule out HSL+ lesions in all such cases. Other authors have proposed the utilization of HRA in screening for these lesions, based on the high frequency of false-negative cytology results in screening for intraepithelial lesions.^[24]^

Finally, the sole predictive factor for HR-HPV anal infection in the multiple logistic regression analysis was a CD4 nadir <200 cells/μL. This factor may also explain the higher rate of HPV infection reported in seropositive versus seronegative MSM patients,^[19]^ their lower capacity to clear these viruses, with a higher incidence of anal mucosa infection^[25,26]^ and faster progression in the severity of precursor lesions.^[27]^ It has been reported that the presence of AIDS is associated with an increased risk of HPV infection-related cancer, finding standardized incidence ratios of 68.6% (95% CI 59.7–78.4) for anal cancer in situ and of 34.6% (95% CI 30.8–38.8) for SSCA.^[27]^ The early diagnosis of HIV patients and minimization of late presentation is therefore vital, because HIV immunosuppression increases the likelihood of acquiring new HPV genotypes and may favor the persistence and reactivation of latent infection by this virus, increasing the risk of these lesions in severely immunodepressed patients.

Study limitations include its single-center design, although it proved possible to enroll all patients in the program, to which all HIV-positive MSM patients in the hospital catchment area were referred. In addition, the results cannot be extrapolated to other types of patient with HIV. The fact that genotyping and histological findings were obtained for all of the patients is a major strength of the present investigation, contrasting with studies in which biopsy was only performed in those with abnormal cytology and with HRA findings of lesions suggestive of dysplasia.

In conclusion, there is a high prevalence of oncogenic HPV infection and HSIL+ lesions in the anal mucosa of HIV-positive MSM. This infection appears to be favored by a deficient immune system, underscoring the need to minimize late diagnoses of HIV. Although HPV 16 was the most prevalent genotype in the anal canal of these patients, the presence of HSIL+ lesions was associated with genotypes 53 and 68.

## Acknowledgment

The authors thank Mercedes Álvarez Romero of the Department of Infectious Diseases for helping to coordinate the patients, assisting with HRA examinations, and drawing blood samples, and to Marina Gutiérrez and Rodrigo López of the Pathology Department for processing samples.
